# Neuroprotective Properties of Melissa Officinalis L. Extract Against Ecstasy-Induced Neurotoxicity

**Published:** 2011-04-21

**Authors:** Gholamreza Hassanzadeh, Parichehr Pasbakhsh, Mohammad Akbari, Saeed Shokri, Mohammadhosein Ghahremani, Gholamreza Amin, Iraj Kashani, Abolfazl Azami Tameh

**Affiliations:** 1. Anatomy Department, School of Medicine, Tehran University of Medical Sciences, Tehran, Iran; 2. Toxicology - Pharmacology Department, Faculty of Pharmacy, Tehran University of Medical Sciences, Tehran, Iran; 3. Herbal Plants Department, School of Pharmacy, Tehran University of Medical Sciences, Tehran, Iran; 4. Anatomy Department, Anatomical Sciences Research Center, School of Medicine, Kashan University of Medical Sciences, Kashan, Iran

**Keywords:** Melissa Officinalis, MDMA, Ecstasy, Neurotoxicity

## Abstract

**Objective:**

The aim of the present study was to investigate the neuroprotective effects of Melissa officinalis, a major antioxidant plant, against neuron toxicity in hippocampal primary culture induced by 3,4-methylenedioxymethamphetamine (MDMA) or ecstasy, one of the most abused drugs, which causes neurotoxicity.

**Materials and Methods:**

3-(4,5-dimethyl-2 thiazoyl)-2,5-diphenyl-tetrazolium bromide (MTT) assay was used to assess mitochondrial activity, reflecting cell survival. Caspase-3 activity assay and Hoechst / propiedium iodide (PI) staining were done to show apoptotic cell death.

**Results:**

A high dose of ecstasy caused profound mitochondrial dysfunction, around 40% less than the control value, and increased apoptotic neuronal death to around 35% more than the control value in hippocampal neuronal culture. Co-treatment with Melissa officinalis significantly reversed these damages to around 15% and 20% respectively of the MDMA alone group, and provided protection against MDMA-induced mitochondrial dysfunction and apoptosis in neurons.

**Conclusion:**

Melissa officinalis has revealed neuroprotective effects against apoptosis induced by MDMA in the primary neurons of hippocampal culture, which could be due to its free radical scavenging properties and monoamine oxidase (MAO) inhibitory effects.

## Introduction

3, 4-methylenedioxymethamphetamine (MDMA) or ecstasy is one of the most abused drugs due to its psychoactive effects (1). Previous studies on the neurotoxic properties of MDMA are focused mostly on the damage of serotonergic and dopaminergic neurons, which are situated mainly in the midbrain (2, 3) However, apoptotic changes in hippocampal neurons in MDMA-treated cultures have also been detected (4). These changes could be due to an oxidative stress event resulting from chronic ecstasy exposure (5). Oxidative stress incidents produce reactive oxygen species (ROS), including hydrogen peroxide which is involved in neurotoxic events related to some neurodegenerative diseases (6). Therefore, extreme production of ROS might cause protein and lipid oxidation leading to neuronal death and apoptosis (7). Natural antioxidants from flora are wellknown to maintain the human organism safe from free radicals and protect it from some diseases (8). It is known that lemon balm or Melissa officinalis L. (Lamiaceae) extracts contain some compounds such as flavonoids and phenolic acids (9) that may scavenge these free radicals and prevent apoptosis. The leaves of Melissa officinalis have some nerve-calming effects according to traditional medicine (10) and are also useful for the improvement of clinical dementia symptoms caused by Alzheimer’s disease (11). The neuroprotective effects of this plant were investigated using an *in vitro* cellular model with the PC12 cell line, which shows some characteristics of neurons (12).

In a previous study, we found that high doses of ecstasy correlate with an increase in caspase-3 activity and apoptosis in primary culture of hippocampal neurons (4). In the present work, we studied whether apoptotic neuronal death induced by ecstasy is abolished by treatment with the antioxidant plant Melissa officinalis.

## Materials and Methods

### Time and setting

Performed at the Department of Anatomy, School of Medicine, and Department of Toxicology-Pharmacology, Faculty of Pharmacy, Tehran University of Medical Sciences, in 2008.

### Chemicals

Buffers, culture plates, and other cell culture materials (except media), rabbit anti-microtubuleassociated protein-2 (MAP2) polyclonal antibody, Hoechst 33342, propiedium iodide (PI), Mowiol 40-88 (324590), caspase-3 colorimetric assay kit and 3- (4, 5-dimethylthiazol-2-yl)-2, 5-diphenyltetrazolium bromide (MTT) were purchased from Sigma (USA). DMEM, Neurobasal medium, FBS, supplement B27 and FITC-goat anti-rabbit antibody were obtained from Invitrogen (Germany). Anthos 2020 microplate reader and fluorescence microscope were respectively from Biochrom (UK) and Nikon (Japan).

### Animals

Sixteen female, pregnant, Wistar rats were obtained from the Razi Vaccine and Serum Research Institute in Karaj, Iran. The rats were housed in a temperature-controlled room (22 ± 2) ℃ and maintained on a 12-hour light/dark cycle with free access to food and water.

All procedures were performed in accordance with institutional guidelines for animal care and use.

### MDMA extraction

MDMA was extracted from ecstasy tablets, which were kindly supplied by the Pharmacology-Toxicology Department, Faculty of Pharmacy, Tehran University of Medical Sciences, Iran, and extracted as described before (13).

### Plant material

Aerial parts of cultivated flowering plant Melissa officinalis L. were collected in 2008 in the Institute of Medicinal Plants (Karaj, Iran) and were confirmed and deposited at the Herbarium of the Department of Herbal Plants, Faculty of Pharmacy, Tehran University of Medical Sciences.

### Preparation of extracts

Dried leaves were ground to a fine powder. The powdered leaves (50g) were macerated in distilled water (500 ml) at room temperature for 24 hours. Subsequently, the mixture was filtered using Watman filter paper. The filtrate was concentrated and lyophilized by freeze drying and then kept in glass vials at -40℃ prior to biological assays. The yield of extract was 15% (w/w).

### Hippocampal neuronal culture

Dissociated hippocampal neurons were prepared from 18-19 day old Wistar rats using a method described previously (14). Briefly, pregnant female rats were anesthetized and killed by cervical dislocation and subjected to caesarean section to obtain fetal brains. Culture method was used with some modifications. Brains were removed from the skull and collected in Hank’s balanced salt solution on ice. The meninges were removed from cerebral hemispheres and the hippocampi were dissected, minced into small pieces, and digested with 0.25% trypsin for 20 minutes at 37 ℃. Fetal bovine serum (FBS) was used to inactivate the trypsin. Finally, the cells were centrifuged for 5 minutes at 900 rpm/minute, resuspended in DMEM with 10% FBS and 500 µm L-glutamine plus antibiotics, and plated in poly-D-lysine hydrobromide (100 µg/ mL)-coated plates. After 1 day, neurobasal medium supplemented with 1% B27 was replaced and half of the medium was exchanged with the new one every 3 days. The cultures were maintained at a temperature of 37 ℃ and 5% CO_2_ and cultured for 7 days prior to treatment. Because the neuronal cultures were serum-free, glia and microglia were virtually absent in the cultures and neuronal purity was assessed by incubation with rabbit anti-MAP2 polyclonal antibody (1: 300 dilution) overnight at 4 ℃, followed by FITC-labeled goat anti-rabbit antibody (1: 1000 dilution) for 1 hour at room temperature, Hoechst 33342 counterstaining (1: 10000 dilution) for 10 minutes, and cover slipping in Mowiol 40-88 for counting.

### Neuronal viability and experimental groups

The (4, 5-dimethylthiazol-2-yl)-2, 5-diphenyl tetrazolium (MTT) assay was used to evaluate the reduction-oxidation status of living cells and mitochondrial activity, reflecting cell survival due to the formation of formazan (15). A density of 1 × 104 cells/well in 96-well plates was used for the MTT assay. Several MDMA (100-1500 µmol/L) and plant extract (10-1000 µg/ml) concentrations were used separately to determine lethal concentration 50 (LC50), which were subsequently determined to be around 1500 µmol/L in MDMA and 100 µg/ml in Melissa. One toxic concentration of MDMA below 1500 µmol/L which was 1200 µmol/L served as MDMA group and one concentration of Melissa below 100 µg/ml, which was 10 µg/ml, served as Melissa group. Neurons which were exposed to both MDMA and Melissa served as MDMA plus Melissa group.

Briefly, neurons were incubated in medium containing 500 µg/mL MTT for 3 hours at 37℃. MTTcontaining medium was removed by plate inversion and 100 µL DMSO was added to each well to dissolve the formazan crystals. The plates were read using an Anthos2020 microplate reader at a wavelength of 570 nm and a reference of 690 nm.

### Caspase-3 activity assay

Neurons (8 × 10^5^ cells/well in 6-well plates) were treated with MDMA, Melissa or MDMA plus Melissa for 24 hours and were assessed for caspase- 3 activity assay. The assay was performed according to a previously described method (16) using the caspase-3 colorimetric assay kit. Briefly, after replacing cell culture medium with caspase lysis buffer, the cell lysates were incubated at 37 ℃ with Ac-DEVD-pNA colorimetric substrate. The amount of P-nitroanilide was continuously monitored over a 60-minute period through the use of a plate reader. Absorbance was measured at 405 nm, normalized to absorbance of control groups, and expressed as percent of control. Each experiment was run in triplicate.

### Propidium iodide/Hoechst staining

Cell death was determined by 4 hours incubation of cultures in medium containing 4 µl/ml propidium iodide (PI) (500µg/ml, Sigma, Germany) before fixation. Viable neurons with cell membrane integration could pump PI out hence late apoptotic and necrotic cells not, here are presented as PI positive neurons. Hoechst 33342 staining (0.1 µg/ml) was done for 10 minutes after fixation in order to normalize PI positive neurons to the total number of nuclei in the field. PI positive neurons were counted under a fluorescence microscope (Nikon, Japan) at an excitation wavelength of 365 (Hoechst) and 530 nm (PI).

### Data analysis

Data were analyzed using SPSS software (version 11.0, Chicago, IL, USA). One-way analysis of variance was used to determine overall significance. Differences between control and experimental groups or between experimental groups were assessed with post-hoc Bonferroni comparison, with significant differences represented as **p<0.01 and *p<0.05 versus control or MDMA group.

## Results

### Neuronal purity

**Fig 1:**
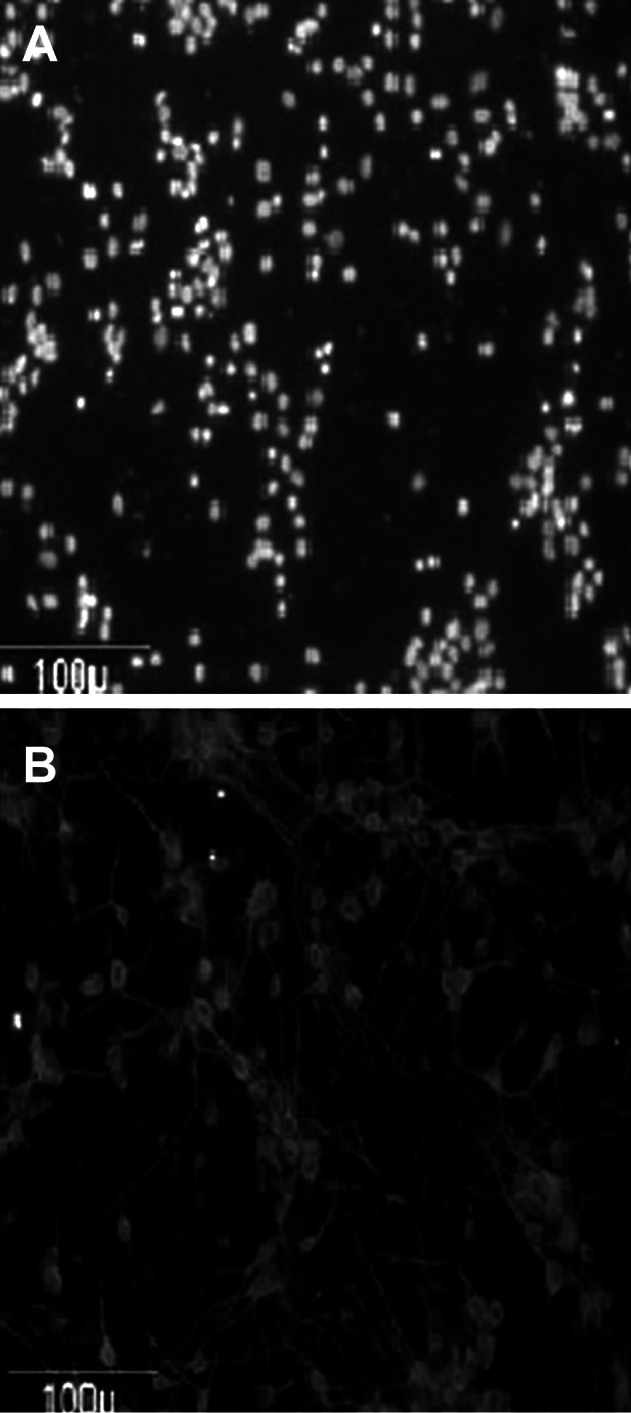
Neuronal purity according to quantification of cells stained with Hoechst (A) and microtubule-associated protein-2-positive neurons (B).

Assessment of neuronal purity was performed using an antibody specific to the neuronal marker MAP2, followed by nuclear counterstaining with Hoechst 33342 dye. Approximately 90% of the cells were MAP2-positive (Fig 1).

### Hippocampal neuronal metabolism and viability

**Fig 2:**
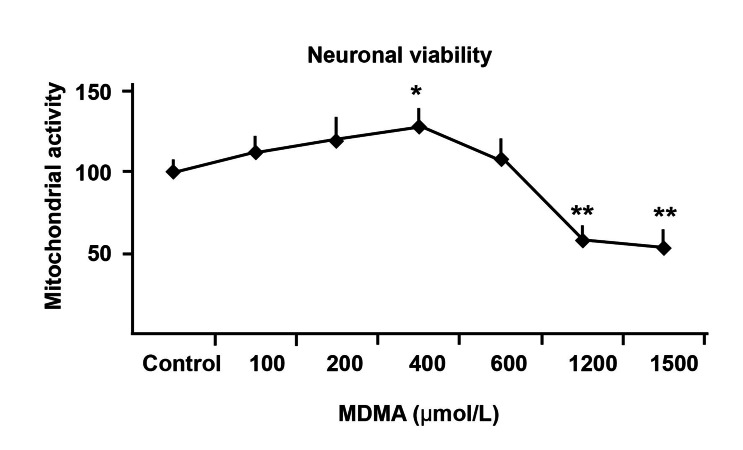
Different concentrations of 3, 4- methylenedioxymethamphetamine (MDMA) were added to wells to obtain a toxic concentration of the drug which was around 1200 µmol/L. So this dose of MDMA was chosen for the rest of the experiments. **p<0.01, vs. control cultures.

**Fig 3:**
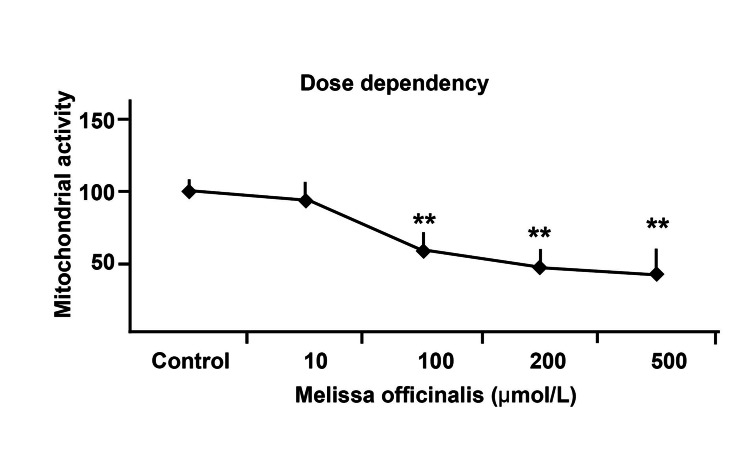
Different concentrations of Melissa officinalis were added in order to find lethal concentration 50. As you see above, the dose of 10 µg/ml has no effect on hippocampal neurons. However, higher than 100 µg/ml decreases cell viability and is toxic for neurons. **p<0.01, vs. control cultures.

**Fig 4:**
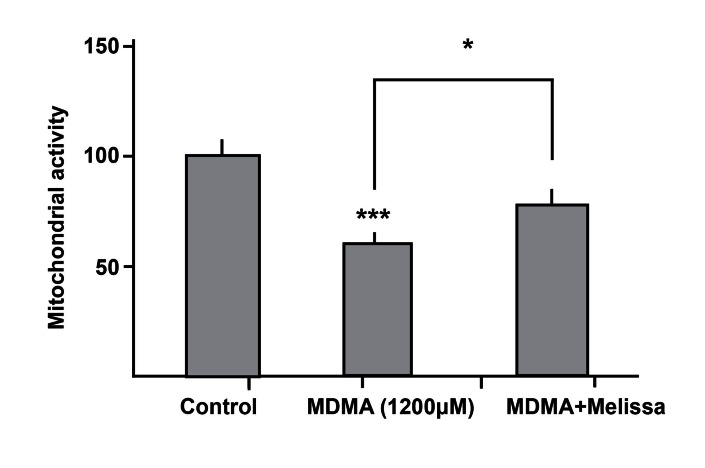
Adding 10µg/ml Melissa to toxic regime of MDMA (1200µM) caused a significant increase in neuronal viability. **p<0.01, vs. control and *p<0.05, vs. MDMA group.

MDMA-induced neurotoxicity in hippocampal neuronal cultures was dose-dependent and at high concentrations (more than 1000 µmol/L), cell viability decreased (Fig 2). 10 µg/ml of Melissa had no effect on hippocampal neuronal cultures although higher concentrations were toxic (Fig 3). There was a significant increase in neuronal viability after using a combination of MDMA and Melissa compared with the MDMA group (Fig 4).

**Fig 5:**
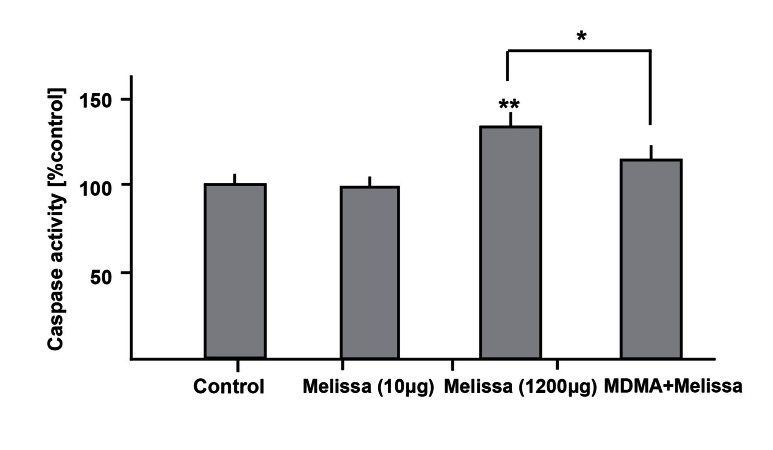
A dose of 1200µM MDMA increases caspase-3 activity and adding 10µg/ml Melissa to this toxic regime caused a significant decrease in caspase-3 activity. **p<0.01, vs. control and *p<0.05, vs. MDMA group.

**Fig 6:**
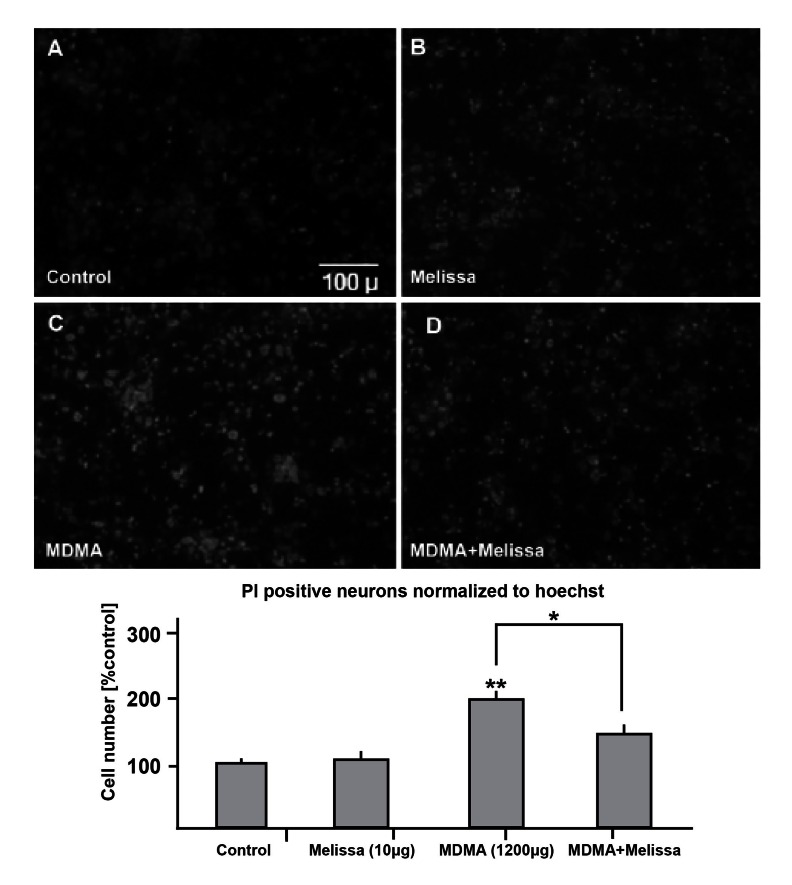
Toxic regimes of MDMA increases PI positive hippocampal neurons (A and C) and adding 10 µg/ml Melissa caused a significant decrease in neuronal death (C and D). Neurons were also treated just with the same dose of Melissa as sham (B). The below graph shows the total number of PI positive neurons which are normalized to Hoechst and quantified as % control. **p < 0.01, vs. control and *p < 0.05, vs. MDMA group.

### Effects of Melissa on MDMA-induced caspase-3

activation in hippocampal neurons Exposure of hippocampal primary neurons to 1200 µmol/L MDMA increased active caspase-3 enzyme compared with the control groups. However, a combination of MDMA and Melissa decreased caspase-3 activity to around 80% of MDMA group value (Fig 5).

### Morphological and apoptotic/necrotic changes after MDMA exposure

A toxic dose of MDMA led to nuclear chromatin condensation showing apoptosis / necrosis in hippocampal neuronal cultures which were stained with Hoechst/PI. This increase in apoptosis/necrosis was significantly reduced in the MDMA plus Melissa treatment group (Fig 6).

## Discussion

The cytotoxicity assay demonstrated that high concentrations of Melissa officinalis aqueous extract decreased neuronal viability. An *in vitro* cytotoxicity assay using the same method as ours also indicated that Melissa is toxic against a series of cancer cell lines (17). Lemon balm has a high percentage of aldehydes which are used as anti-infectious agents (18). Elevated amounts of these agents in high doses of Melissa could be the main cause of decline in neuronal viability, although another study is running in order to establish an exact dose response of this plant extract in the hippocampi of rats *in vitro* (Unpublished).

In the present study low concentrations of MDMA increased mitochondrial activity of hippocampal primary cultures, resembling cell viability, and conversely high doses of it lowered neuronal viability leading to apoptosis which is approved by caspase-3 activity assay and was discussed in our previous study (4). This event is attenuated by treatment with 10 µg/ml of Melissa officinalis to around 20% of MDMA group. A previous study using the PC12 cell line which resembles some characteristics of neurons, reported increase in cell survival after pretreatment with both aqueous and methanolic extract of Melissa against H_2_O_2_ toxicity (12). The aqueous extract of Melissa contains mainly water-soluble compounds such as phenolic acids (rosmarinic acid) (17) which have a strong antioxidant effect although the same effect from methanolic extract has also been proven (12) and more studies are needed to determine the exact fraction/s of this plant in cell survival. Considering that oxidative stress is induced by high doses of MDMA in hippocampi (5), we can assume that the neuroprotective effects of Melissa in the MDMA plus Melissa group arise from its free radical scavenging properties. However, we could not show this procedure by fractionation of Melissa extract or find the best fraction to be used for this protection. The protective effect of Melissa in neuronal viability was in agreement with the results of caspase-3 activity and PI staining assessments. As reported before, high doses of MDMA induced apoptosis in hippocampal primary culture and 10 µg/ml of Melissa caused a significant 20% reduction of caspase-3 activity compared to the MDMA group. Caspase-3 is a key element in many apoptotic pathways and the termination of this process has been confirmed? by PI staining. Therefore, reduced caspase-3 expression and PI positive neurons in the MDMA plus Melissa group could lead to neuronal survival. Another possible factor in the neuroprotective quality of Melissa could be its monoamine oxidase (MAO) inhibition (12) which may interfere with the monoamine transporter inhibition property of MDMA (19).

## Conclusion

Melissa officinalis has revealed neuroprotective effects against apoptosis induced by MDMA in the primary neurons of hippocampal culture which could be due to its free radical scavenging properties and MAO inhibitory effects.

These results propose the potential use of this plant for central nervous system disorders and as a neuroprotective agent to prevent neurodegenerative diseases, although more research has to be done in order to determine the exact fraction of Melissa and the molecular mechanisms involved in this neuroprotection.
